# Patient-reported common symptoms as an assessment of interventions in medication reviews: a randomised, controlled trial

**DOI:** 10.1007/s11096-017-0575-7

**Published:** 2017-12-05

**Authors:** Tim W. A. Schoenmakers, Michel Wensing, Peter A. G. M. De Smet, Martina Teichert

**Affiliations:** 1 Department of IQ Healthcare, Radboud Institute for Health Sciences, Radboud University Medical Center, PO Box 9101, 6500 HB Nijmegen, The Netherlands; 2 Zorgapotheek Nederland BV, Utrecht, The Netherlands; 3 Department of General Practice and Health Services Research, University Hospital Heidelberg, Heidelberg, Germany; 4 Department of Clinical Pharmacy, Radboud Institute for Health Sciences, Radboud University Medical Center, Nijmegen, The Netherlands; 5 Department of Clinical Pharmacy and Toxicology, Leiden University Medical Center, Leiden, The Netherlands

**Keywords:** Adverse drug events, Community pharmacies, Medication review, Patient-reported outcomes, Pharmaceutical care, PROMISE, Side effects, Netherlands

## Abstract

*Background* A ‘Patient-Reported Outcome Measure, Inquiry into Side Effects’ (PROMISE) instrument was developed for patients to report common symptoms in clinical medication reviews. *Objective* To determine changes in patient-reported drug-associated symptoms collected by PROMISE before and after community pharmacist-led clinical medication reviews compared with usual care. *Setting* Community pharmacies in the Netherlands. *Methods* Patients were randomised into an intervention group (IG) and a control group (CG). PROMISE was used to collect symptoms experienced during the previous month, and any suspected drug-associated symptoms from both groups at baseline and at follow-up after 3 months. IG patients received a one-time clinical medication review, while CG patients received usual care. *Main outcome measure* Mean number of drug-associated symptoms at follow-up. *Results* Measurements were completed by 78 IG and 67 CG patients from 15 community pharmacies. Mean numbers of drug-associated symptoms per patient at follow-up were 4.0 in the IG and 5.0 in the CG, with an incident rate ratio between the groups of 0.90 (95% CI 0.62–1.33). Mean numbers of drug-associated symptoms per patient reported at follow-up and persisting since baseline were 2.1 in the IG and 2.6 in the CG, with an incident rate ratio of 0.85 (95% CI 0.43–1.42). The lowest percentages of persisting drug-associated symptoms detected in the IG were ‘palpitations’ and ‘stomach pain, dyspepsia’ while in the CG they were ‘stomach pain, dyspepsia’ and ‘trembling, shivering’. *Conclusion* PROMISE provided meaningful information on drug-associated symptoms in clinical medication reviews, however the number of drug-associated symptoms was not reduced by performing clinical medication reviews compared with usual care.

**Electronic supplementary material:**

The online version of this article (10.1007/s11096-017-0575-7) contains supplementary material, which is available to authorized users.

## Impacts on practice

- The PROMISE instrument as a patient-reported symptom list is helpful in detecting drug-associated symptoms experienced by patients with multiple drugs in chronic use.
- The integration of PROMISE into the context of a clinical medication review may be useful to detect and improve drug-associated symptoms experienced by patients.

## Introduction

Beside their desired effects, drugs can cause adverse symptoms. Health professionals mainly pay attention to potentially serious drug-related symptoms to prevent major harms to patients [1–4]. In contrast, potentially severe drug-related symptoms tend to be neglected or acknowledged by practitioners as inevitably connected to the drug effect [5–7]. Nevertheless, such common symptoms could substantially impact patient quality of life. For example, muscle pain may reduce physical activity [8] and diarrhoea may impact social activities [9]. Thus, strategies to detect and reduce common drug-related symptoms are needed.

The reduction of drug-related symptoms is one of the aims in a Clinical Medication Review (CMR), which is a structured evaluation of a patient‘s medicines based on medication history, patient information and clinical data [10, 11]. While studies on CMRs have focused on various outcomes, such as drug therapy-related problems (DTPs) [12–14], patient-reported outcomes, such as bothersome common symptoms, are underrepresented [15]. Some studies included patient-suspected drug-related symptoms [16, 17], although only Sorensen et al. [17] identified these by patient reporting through open-ended questioning. However, identification by means of a list of specific symptoms may be preferable as this has higher sensitivity [18].

Similarly to DTPs, patient-reported outcome measures (PROMs) could potentially be used as outcome measures for CMRs, as is done in other areas of clinical research [19]. In the context of patient-centred care, PROMs could prompt a discussion between patients and healthcare professionals on important issues for patients and their healthcare needs [20]. Specifically, information on a patient’s beliefs and concerns about and the use of his/her drugs could be collected to optimise and individualise drug treatment [21]. Thus the ‘Patient-Reported Outcome Measure, Inquiry into Side Effects’ (PROMISE) instrument was developed to facilitate the gathering of meaningful information from patients in CMRs to support pharmacists in detecting drug-related symptoms [22], and to use this information as PROMs in CMRs. In this trial the ability of the instrument to detect changes was assessed.

## Aim of the study

The aim of this study was to determine changes in patient-reported drug-associated symptoms collected by PROMISE before and after community pharmacist-led CMRs compared with usual care.

## Ethics approval

The Arnhem-Nijmegen medical ethical committee waived ethical approval for this study (registration number 2014-320).

## Methods

### Study design and setting

This non-blinded, randomised, controlled trial was conducted in the Netherlands (registered in the Netherlands Trial Register under number 4895, www.trialregister.nl). Patients eligible for a CMR according to the guidelines were invited to participate in the study by their community pharmacist [23, 24]. After providing written informed consent, patients were randomised into an intervention group (IG) and a control group (CG). On average 13% of the patients in the Netherlands use five or more drugs long term [25]. In the Netherlands, community pharmacists collect patients’ medication history in information systems. These data are used for regular medication surveillance, and for performing CMRs combined with patient information and clinical information. At present, community pharmacists have to conduct a specified number of CMRs annually for susceptible elderly on behalf of the Dutch Healthcare Inspectorate, which are reimbursed by the health insurance companies. The performance of CMRs is defined in guidelines and comprises six steps for patient selection, patient interview, medication analysis, intervention plan, implementation of treatment changes, and evaluation after 3 months of follow-up; the patient and the general practitioner (GP) also contribute to these steps [23, 24].

### Interventions

#### PROMISE

PROMISE was developed as a paper-based instrument to collect patient information in six domains for the patient interview in CMRs and for the follow-up evaluation (Online Resource 1). The main domain comprised 22 common predefined symptoms, with the option of reporting additional symptoms [22]. Patients were asked to report all symptoms experienced in the previous month (yes/no) and to report any suspicion that these symptoms were associated with the drugs they were using (yes/do not know/no). Symptoms with the answer ‘yes’ or ‘do not know’ in the second component were further evaluated as drug-associated symptoms (DAS). In PROMISE, additional information was collected in four other domains based on existing validated instruments: general health perception [7], a question about self-rated health that can serve as a global measure of health status [26, 27]; necessity and concern beliefs, five of the ten items in the Beliefs about Medicines Questionnaire reflecting the current and future necessity and concern beliefs, and the concern beliefs about the knowledge of the medicines [28]; self-efficacy in understanding and using medicines, one item for both subscales of the eight-item MUSE scale [29]; medication adherence from the patient’s perspective according to the frequently used Medication Adherence Rating Scale [21, 30–32]. Finally, patients could propose other issues to be discussed in the interview with the pharmacist [22].

### Intervention and usual care

For the IG, the completed PROMISE instrument at the start of the CMR was used to identify DTPs, such as patient-reported DAS, and to collect additional information relevant to the patient’s drug use. During the patient interview in the CMR, the answers to the PROMISE instrument were discussed between the patient and pharmacist. The information on DAS was used by pharmacists to elucidate the burden for the patient, assess the potential cause, and decide on an intervention regarding drug therapy [23, 33]. With this information, the following steps of the CMR were followed to agree on an improved treatment plan together with the GP and patient. The CG completed PROMISE without a subsequent CMR. After 3 months of follow-up, PROMISE was repeated by the IG and CG.

### Pharmacists

All participating pharmacists were invited from pharmacists affiliated to ‘Pluriplus’, a Dutch pharmaceutical care support organisation providing an online tool called ‘Nexus Medication Check’ which facilitates the implementation of medication reviews. All participating pharmacists were already trained and experienced in performing CMRs according to the Dutch guidelines for CMR [18, 19]. The pharmacists received written and oral instructions for sampling of patients, using PROMISE in the CMR, and collecting study data.

### Patients

Patients were eligible for study inclusion if they met the guideline-based inclusion criteria for CMRs [24]. First, chronic use of at least five drugs was determined by means of the online tool. Subsequently, further sampling was applied by pharmacists based on additional risk factors like age over 65 years, comorbidities, decreased adherence, and use of risk medication. Finally, pharmacists applied additional criteria such as cooperation with the relevant GP. Patients were excluded if they met one of the following criteria: cognitive impairment, personal or health issues hindering participation according to the GPs; recent participation in other pharmacotherapy interventions; unwilling or unable to participate in a CMR according to the pharmacist. Pharmacists recruited patients by telephone or by mail, aiming to form a group of 20 participants. All patients in a pharmacy who provided written informed consent were randomised into the IG or CG by a research collaborator in blocks of four patients using computer-generated code lists.

### Outcomes

The primary outcome was the mean number of DAS at follow-up, as measured with PROMISE in the IG compared with the CG.

Additional outcomes were the mean number and types of DAS reported at baseline that persisted at follow-up as well as the number of patients who reported at least one DAS at follow-up in the IG compared with the CG. Furthermore, for persisting DAS a number needed to treat (NNT) was calculated to express the number of patients needed to participate in the intervention to solve one additional DAS. Additionally, the patient-reported scores of the other domains of PROMISE were measured as outcomes at follow-up: health perception, necessity beliefs (mean of two items), concern beliefs (mean of three items), self-efficacy (mean of two items), and medication adherence (sum of five items).

### Sample size calculation

We designed the trial to detect a provisionally estimated difference of 10% in the number of DAS between the IG and CG at follow-up. With an alpha of 5%, a power of 80%, and a intraclass correlation coefficient of 0.05 to correct for potential dependencies within a pharmacy a number of 90 subjects per group were needed. Assuming a drop-out rate of 10% during follow-up we aimed to enrol 100 patients per group, giving a total of 200 subjects.

### Data collection

The pharmacists sent the completed PROMISE and other documents with anonymised information on actual drug use and patient sex and age to the researchers. All dispensed drugs covering the month before completing PROMISE were considered to be in use and were recorded [22] according to the 2013 version of the Anatomic Therapeutic Chemical classification system of the World Health Organisation [34].

### Statistical analysis

All data from PROMISE, the actual medication status and the interview protocols were recorded in a Microsoft Access database, version 2007 (Microsoft Corp., Redmond, WA) and were analysed with SPSS version 22 (IBM Corp., Armonk, NY). All patients who completed PROMISE at baseline and follow-up were included for analysis. Descriptive statistics were applied to patient characteristics, drugs in use, and data from PROMISE. In a sensitivity analysis only symptoms reported by patients with certainty as DAS (yes) were used.

### Primary outcome

A negative-binomial log linear regression model was built to assess differences between the IG and CG in mean numbers of DAS per patient at follow-up, expressed as incident rate ratios (IRR) where an IRR of 1 signifies no difference. Differences were adjusted for mean number of DAS at baseline and potential confounders (sex, age and number of drugs in use at baseline) were added to the model. If a patient clustering effect per pharmacy was detected, differences in primary outcome were assessed using mixed model regression analysis with negative-binomial distribution and log link function.

### Additional outcomes

Differences between the IG and CG in mean number of DAS persisting at follow-up per patient adjusted for mean number of DAS at baseline (expressed as IRR) were assessed with a negative-binomial log linear regression model. A logistic regression model was used to assess differences between numbers of patients reporting a specific DAS persisting at follow-up, adjusted for differences in numbers of patients reporting that particular DAS at baseline. The NNT for persisting DAS was calculated as the inverse of the absolute risk reduction, where the absolute risk reduction was defined as the difference between the CG event rate and the IG event rate; these event rates were expressed as the number of persisting DAS at follow-up divided by the number of DAS at baseline.

For general health perception, the scores were dichotomised; the patient scores ‘very good’ and ‘good’ were considered as ‘healthy’, and ‘fair’ (‘relatively healthy’), and ‘bad’ or ‘very bad’ (‘unhealthy’) were considered as ‘other’ [26]. For self-efficacy a mean score was calculated only for patients who answered at least two items. The mean scores were dichotomised as follows: a mean score of four (totally agree) was considered as ‘good’, while all other mean scores were considered as ‘other’. For the Medication Adherence Rating Scale, a sum score ranging from 5 to 25 was calculated only for patients who answered all items. The sum score was dichotomised, and a sum score of 22 or lower was considered as non-adherent according to earlier studies [21, 35]. For these domains, differences in scores between the IG and CG at follow-up (adjusted for differences at baseline) were assessed using logistic regression analysis.

For necessity beliefs (two items) and concern beliefs (three items), means were calculated only for patients who answered at least two items. Differences in mean scores between the IG and CG at follow-up were assessed using a linear regression model adjusted for differences at baseline.

Potential confounders (sex, age and number of drugs in use at baseline) were added to the model for all additional outcomes. When an effect of a pharmacy for patient clustering was detected for a specific outcome, differences were assessed using the corresponding mixed regression model analysis.

## Results

**Fig. 1:**
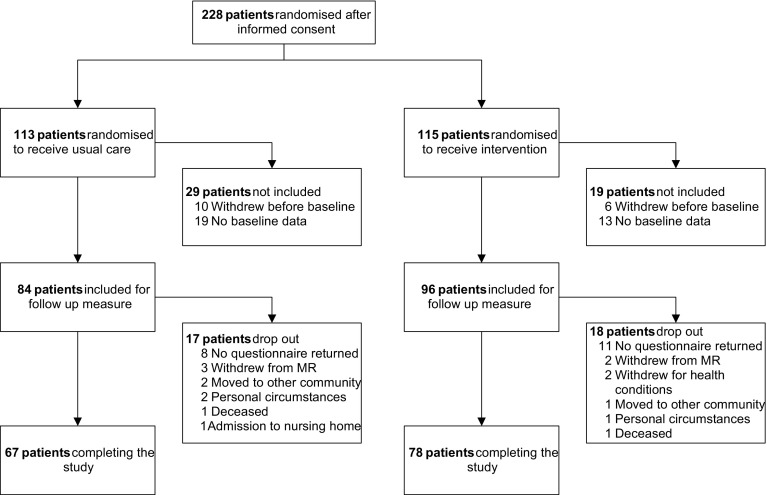
Flowchart

A total of 228 patients from 15 community pharmacies provided informed consent and were invited to participate in the study between September 2014 and October 2015. Patient numbers per pharmacy varied from 6 to 29 included patients and 4 to 24 patients who completed PROMISE at baseline and follow-up. Information from 48 patients could not be included due to withdrawal after randomisation or incomplete baseline data. From the remaining 180 participants, 145 (80.6%) patients completed the measurement at follow-up between January 2015 and June 2016 (78 in the IG and 67 in the CG) (Fig. 1). Of these patients, 53.1% were female, and the mean age was 73 years (range 49–89) (Table 1). The IG and CG did not differ in sex, age, mean numbers and drug classes in use. The types of most frequently reported DAS at baseline were comparable in both groups, except for ‘dry mouth, thirst, mouth complaints’ and ‘muscle pain, joint pain’, which were reported less often in the CG.

**Table 1:** Baseline patient characteristics

	Intervention N = 78	Control N = 67
Number of females (%)	40 (51.3)	37 (55.2)
Mean age [years (range)]	73.3 (49–88)	72.6 (49–89)
Mean number of drugs in use (range)	8.7 (5–22)	9.5 (5–20)
Number of drugs in use [number of patients (%)]		
5–7	29 (37.2)	25 (37.3)
8–10	35 (44.9)	18 (26.9)
> 10	14 (17.9)	24 (35.8)
Most frequently used drug classes (ATC code) [number of patients (%)]		
Proton pump inhibitors (A02BC)	55 (70.5)	42 (62.7)
HMG CoA reductase inhibitors (C10AA)	50 (64.1)	41 (61.2)
Beta blocking agents, selective (C07AB)	44 (56.4)	38 (56.7)
Platelet aggregation inhibitors excl. Heparin (B01AC)	33 (42.3)	30 (44.8)
ACE inhibitors, plain (C09AA)	30 (38.5)	24 (35.8)
Angiotensin II antagonists, plain (C09CA)	22 (28.2)	27 (40.3)
Thiazides, plain (C03AA)	23 (29.5)	24 (35.8)
Dihydropyridine derivatives (C08CA)	20 (25.6)	23 (34.3)
Vitamin K antagonists (B01AA)	18 (23.1)	18 (26.9)
Biguanides (A10BA)	17 (21.8)	16 (23.9)
1. Perceived health: number of ‘healthy’ patients^a^ (%)	37 (47.4)	30 (44.8)
2a. Necessity beliefs, mean score per patient (SD)^b^	3.71 (0.78)	3.95 (0.80)
2b. Concern beliefs, mean score per patient (SD)^c^	2.56 (0.71)	2.72 (0.73)
3. Number of patients reporting ‘good’ self efficacy (%)^d^	36 (46.2)	36 (53.7)
4. MARS, number of patients non-adherent (%)^e^	11 (14.1)	10 (14.9)
Mean number of symptoms reported	5.8	6.0
Mean number of DAS reported	5.1	4.8
Number of DAS reported [number of patients (%)]		
0	16 (20.5)	12 (17.9)
1–2	13 (16.7)	13 (19.4)
3–4	13 (16.7)	14 (20.9)
5–7	19 (24.4)	13 (19.4)
> = 8	17 (21.8)	15 (22.4)
Most reported DAS within study population [number of patients (%)]		
Dry mouth, thirst, mouth complaints	32 (41)	18 (23)
Muscle pain, joint pain	33 (42)	23 (29)
Weakness, tiredness	29 (37)	27 (35)
Bruises, bleedings	24 (31)	26 (33)
Skin complaints, itching	25 (32)	24 (31)
Flatulence	26 (33)	24 (31)

*MARS* The Medication Adherence Rating Scale; *DAS* drug-associated symptoms

^a^Patients reporting ‘very good’ or ‘good’ are considered healthy

^b^Mean of two items, for each item: 1 = totally disagree, 5 totally agree; higher scores on scale indicate higher beliefs of necessity

^c^Mean of three items, for each item: 1 = totally disagree, 5 totally agree; reverse scored so lower scores on scale indicate less concerns

^d^Mean score of 4 (totally agree) from two items was considered as ‘good’ self-efficacy

^e^A sum score of five items (for each item: 1 = always, 5 = never) < = 22 was considered as non-adherent

The mean numbers of DAS per patient at follow-up were 4.0 in the IG (5.1 at baseline) and 5.0 in the CG (4.8 at baseline) (Table 2). The IRR between the IG and CG was 0.90 [95% confidence interval (CI) 0.62–1.33], implying a higher reduction in the IG. In the sensitivity analysis, mean numbers of DAS (answer ‘yes’) per patient at follow-up were 1.0 in the IG (1.4 at baseline) and 1.8 in the CG (2.4 at baseline), and the IRR for the mean numbers of DAS (answer ‘yes’) between the IG and CG was 0.72 (95% CI 0.45–1.15).

**Table 2:** Mean number of drug-associated symptoms at follow-up

	Intervention N = 78	Control N = 67	Differences between groups (IG vs. CG)
DAS, mean number per patient (total number) (*primary outcome*)	4.0 (313)	5.0 (333)	Incidence rate ratio 0.90; 95% CI 0.62–1.33^a^
DAS persisting at follow-up, mean number per patient (total number)	2.2 (171)	2.6 (174)	Incidence rate ratio 0.85; 95% CI 0.55–1.30^a^
Number of patients reporting at least one DAS (%)	56 (72)	51 (76)	Odds ratio 0.85; 95% CI 0.38–1.88^b^

*DAS* drug-associated symptoms, *IG* intervention group, *CG* control group, *CI* confidence interval

^a^Negative-binomial log linear regression analysis adjusted for number of DAS at baseline, sex, age and number of drugs in use at baseline

^b^Logistic regression analysis adjusted for differences at baseline, sex, age and number of drugs in use at baseline

The mean numbers of *persisting* DAS per patient at follow-up were 2.1 in the IG and 2.6 in the CG, which meant a reduction for both groups compared with baseline measurements (5.1 and 4.8, respectively). The incidence rate ratio between the IG and CG was 0.85 (95% CI 0.43–1.42) (Table 2). For the persisting DAS, the NNT was nine, implying that nine patients had to receive a CMR with PROMISE to solve one persisting DAS at follow-up.

The percentage of persisting DAS, reported at baseline and again at follow-up, was 43% in the IG and 54% in the CG. For separate symptoms, the percentage of persisting DAS reported by at least 10 patients at baseline varied at follow-up from 11 to 89% (Table 3).

**Table 3:** Number of drug-associated symptoms at baseline and percentage of these persisting at follow-up

	Intervention (N = 78)		Control (N = 67)	
	T = 0	T = 1	T = 0	T = 1
	DAS	% Baseline DAS	DAS	% Baseline DAS
Muscle pain, joint pain	33	52	23	61
Dry mouth, thirst, mouth complaints	32	63	18	89
Weakness, tiredness	29	59	27	56
Flatulence	26	54	24	38
Drowsiness	25	44	18	56
Skin complaints, itching	25	60	24	50
Bruises, bleedings	24	42	26	65
Dizziness, vertigo, fainting	20	20	14	57
Eye irritation, vision problems	19	53	20	50
Constipation	16	19	12	50
Headache	16	44	6	33
Sweating	15	33	12	50
Stomach pain, dyspepsia	14	14	10	30
Diarrhoea	13	38	11	73
Palpitations	12	8	7	43
Trembling, shivering	12	42	11	36
Abdominal pain	11	55	8	50
Change of mood	11	18	13	54
Muscular weakness	10	30	13	46
Nausea, vomiting	9	22	3	33
Sexual complaints	9	67	10	50
Change of appetite	7	14	7	57

*DAS* drug-associated symptoms, *IG* intervention group, *CG* control group

The total number of patients who reported at least one DAS at follow-up was 56 (72%) in the IG and 51 (76%) in the CG. The IG was 15% less likely to report at least one DAS at follow-up; however, this difference was not statistically significant [odds ratio (OR) 0.85; 95% CI 0.38–1.88] (Table 2).

Of the other domains in the PROMISE instrument only ‘self-efficacy’ showed a statistically significant improvement in the IG compared with the CG. More patients in the IG reported ‘good’ self-efficacy at follow-up compared with the CG (OR 2.91, 95% CI 1.20–7.06).

## Discussion

Our study did not show a statistically significant reduction in numbers of DAS at follow-up for patients participating in a CMR with the PROMISE instrument compared with those receiving usual care. However, there might be a potential benefit for the use of PROMISE in CMRs in reducing DAS, as on average one DAS within nine patients participating in a CMR was resolved.

### Absence of reduction in drug-associated symptoms

Our findings are in line with the earlier results of Sorensen et al. [17] who also did not find a statistically significant reduction in patient-reported drug-related symptoms after a CMR. However, it might be difficult to detect specific effects of a complex intervention in complex cases. First, patients eligible for CMRs are likely to consult more healthcare practitioners than patients who are ineligible for CMRs as they are often affected by a range of diseases or minor illnesses. The reported DAS could be manifestations of symptoms related to these ailments. Second, some DAS, such as stomach pain or constipation, may already have been resolved as part of usual care [36]. Third, some side effects, such as headache caused by dihydropyridin derivatives, may be transient in nature and thus resolve without intervention [37]. Finally, the structured questioning may have increased the awareness of DAS among CG patients, which may have encouraged them to act. All these aspects may explain the notable decrease in persisting DAS in the CG at follow-up (from 4.8 to 2.6).

### Increase in self-efficacy

Of the other domains in PROMISE, only self-efficacy in using medication showed a positive effect of the CMR compared with usual care. This may be because a pharmacist can easily improve a patient’s ability to use a drug by providing additional instructions; however, uniform registration of pharmacists’ interventions are needed to confirm this. Furthermore, the literature indicates an association between concern beliefs and patient-reporting of a DAS [38], but this could not be confirmed due to our small sample size.

### Potential benefit of PROMISE in practice

Although using PROMISE within CMRs had no detectable effect on the number of DAS at follow-up, we believe that the PROMISE instrument could be useful to draw the attention of healthcare professionals to common DAS as symptom management is a cornerstone of care for patients with chronic conditions [39]. Furthermore, Willeboordse et al. [40] reported that except for vulnerable patients (characterized by > 4 chronic diseases, > 10 drugs used, and low health literacy) a questionnaire may be as effective as an interview in determining the patient perspective. Hence, the use of PROMISE may improve the feasibility of a CMR as a replacement for or in support of the patient interview.

### Limitations

Our study was not without limitations. First, the number of participants was smaller than intended. In practice, it was difficult to achieve the targeted number of 20 patients per pharmacy. The extra informed consent and randomisation step complicated the usual procedure involved in inviting patients to participate in a CMR. The study period was prolonged in an attempt to reach a sufficient number of participants, but sufficient numbers could not be reached within the frame of the study. The achieved sample size may have been insufficient to prove a possible effect. Second, to reduce additional work for the pharmacists, instructions for pharmacists’ registrations (e.g. the evaluation and follow up actions of DAS), were kept to a minimum; however, this also reduced the possibility to evaluate the plausibility of DAS and potential follow-up actions. A variation in follow-up actions on DAS may be plausible as guidelines on interventions are lacking. Furthermore, pharmacists’ recommendations also have to be accepted by GPs and patients, which is likely to vary between patients and settings [41]. Finally, the pharmacists had little experience with PROMs which may have hindered potential outcomes.

## Conclusion

The PROMISE instrument provided meaningful information on DAS in CMRs; however, the number of DAS was not reduced by the application of CMRs compared with usual care. Further research with larger numbers of patients is needed to investigate the factors that can facilitate the use of PROMISE as a tool to effectively deal with common DAS.

## Electronic supplementary material

Below is the link to the electronic supplementary material.

Supplementary material 1 (PDF 220 kb)
