# Supplementary material for: Patient-reported common symptoms as an assessment of interventions in medication reviews: a randomised, controlled trial
Source: Int J Clin Pharm. 2017 Dec 5;40(1):126–34. doi: 10.1007/s11096-017-0575-7 (PMC5840243; doi:10.1007/s11096-017-0575-7)
Supplement: Supplementary file 1 — Supplementary material 1 (PDF 220 kb) [file 11096_2017_575_MOESM1_ESM.pdf]

Online resource 1: PROMISE Instrument

Patient-reported common symptoms as an assessment of interventions in medication reviews: a randomised, controlled trial

International Journal of Clinical Pharmacy

Tim WA Schoenmakers, PharmD[1,2], Michel Wensing, PhD[1,3], Peter AGM De Smet, PhD[1,4]
Martina Teichert, PhD[1,5]

1. Radboud university medical center, Radboud Institute for Health Sciences, Department of IQ Healthcare, PO Box 9101, 6500 HB Nijmegen, The Netherlands.
   Email: tim.schoenmakers@radboudumc.nl
2. Zorgapotheek Nederland BV, Utrecht, The Netherlands
3. University Hospital Heidelberg, Department of General Practice and Health Services Research, Heidelberg, Germany
4. Radboud university medical center, Radboud Institute for Health Sciences, Department of clinical pharmacy, Nijmegen, The Netherlands
5. Department of Clinical Pharmacy & Toxicology, Leiden University Medical Center, Leiden, The Netherlands

Questionnaire medication review

For the questions below, please mark the boxes that most closely resemble your situation.

1. How would you rate your general health?

very good	good	Fair	bad	very bad
❑	❑	❑	❑	❑

2. What do your medications mean to you?

Can you indicate to what extent you agree or disagree with the following statements (based on **all medications** you used **for the last 3 months,** daily or weekly)?

	strongly disagree	disagree	uncertain	agree	strongly agree
2a. My health at present depends on my medicines	❑	❑	❑	❑	❑
2b. My health in the future will depend on my medicines	❑	❑	❑	❑	❑
2c. My medicines are a mystery to me.	❑	❑	❑	❑	❑
2d. I sometimes worry about the long-term effects of my medicines	❑	❑	❑	❑	❑
2e My medicines disrupt my life.	❑	❑	❑	❑	❑

3. How would you rate your own medicine use?

(based on **all medications** you used **for the last 3 months,** daily or weekly)

	strongly disagree	disagree	agree	strongly agree
3a. I **know** how to take my medications according to the instructions.	❑	❑	❑	❑
3b. I **can** take my medications according to the instructions.	❑	❑	❑	❑

4. How do you use your own medications?

Some people use their medications in their own way. This may differ from the instructions on the label or from what the doctor said. Can you indicate how often each each statement applies to you (based on **all medications** you used **for the last 3 months** daily or weekly)?

	always	often	some-times	rarely	never
4a. I forget to take my medications.	❑	❑	❑	❑	❑
4b. I alter the dose of my medications.	❑	❑	❑	❑	❑
4c. I stop taking my medications for a while.	❑	❑	❑	❑	❑
4d. I decide to miss out a dose.	❑	❑	❑	❑	❑
4e. I take less than instructed.	❑	❑	❑	❑	❑

Suffering from one of the following symptoms

To what extent did you suffer from one of the following symptoms **in the last month?** Can you indicate whether you think that this may be a side effect of one of your medications.

	I **suffered from** the following symptom last month		This symptom was possibly a **side effect** of one of my medications.		
**Symptom**	**yes**	**no**	**yes**	**no**	**do not know**
Change of appetite	❑	❑	❑	❑	❑
Dry mouth/ thirst, mouth complaints	❑	❑	❑	❑	❑
Nausea, vomiting	❑	❑	❑	❑	❑
Stomach pain, dyspepsia	❑	❑	❑	❑	❑
Abdominal pain	❑	❑	❑	❑	❑
Diarrhoea	❑	❑	❑	❑	❑
Constipation	❑	❑	❑	❑	❑
Flatulence	❑	❑	❑	❑	❑
Eye irritation, vision problems	❑	❑	❑	❑	❑
Palpitations	❑	❑	❑	❑	❑
Trembling, shivering	❑	❑	❑	❑	❑
Muscle pain, joint pain	❑	❑	❑	❑	❑
Muscular weakness	❑	❑	❑	❑	❑
Headache	❑	❑	❑	❑	❑
Dizziness, vertigo, fainting	❑	❑	❑	❑	❑
Weakness, tiredness	❑	❑	❑	❑	❑
Drowsiness	❑	❑	❑	❑	❑
Change of mood	❑	❑	❑	❑	❑
Sexual complaints	❑	❑	❑	❑	❑
Bruises, bleedings	❑	❑	❑	❑	❑
Skin complaints, itching	❑	❑	❑	❑	❑
Sweating	❑	❑	❑	❑	❑
Other: ………………………………………………	❑	❑	❑	❑	❑

5. What do you want to discuss with your pharmacist?

Your answers given in this instrument support your pharmacist in discussing your drug use. Do you have any issues, you want to discuss with your pharmacist yourself, whether or not linked to above items?

……………………………………………………………………………………………….

KNMP

IQ Scientific Center for Quality of Healthcare
